# Baits for Suicide.—"Lady Audley's Secret" and "Aurora Floyd"

**Published:** 1863-10

**Authors:** 


### MEDICAL CRITIC

AND

### PSYCHOLOGICAL

### JOURNAL.

OCTOBER, 1863.

### Art. I.—BAITS FOR SUICIDE.—"LADY AUDLEY'S SECRET" AND "AURORA FLOYD."

There is a peculiarity in the two well-known novels, *Lady Audley's Secret* and *Aurora Floyd*, which has not hitherto received the attention it deserves. The peculiarity in question is the manner in which the authoress plays with the idea of suicide.

George Talboys, on board the *Argus*, homeward bound from Australia, tells Miss Morley the story of his marriage, his ruin, his desertion of wife and child. He tells of his wife's stormy grief when first she learnt the exhaustion of their means of livelihood, and how her tears and reproaches drove him almost mad.—"I flew into a rage with her, myself, her father, the world, and everybody in it," he says; "and then ran out of the house declaring that I would never enter it again. I walked about the streets all that day half out of my mind, and with a strong inclination to throw myself into the sea, so as to leave my poor girl free to make a better match. 'If I drown myself, her father must support her,' I thought; 'the old hypocrite could never refuse her a shelter, but while I live she has no claim on him.' I went down to a rickety old wooden pier, meaning to wait there till it was dark, and then drop quietly over the end of it into the water; but while I sat there smoking my pipe, and staring vacantly at the sea-gulls, two men came down, and one of them began to talk of the Australian diggings". . . . (L. A. S. i. 40). And so our hero, his thoughts diverted into another channel, instead of casting himself into the sea, simply casts away his wife and new-born infant. He hies to Australia, and for three years all trace of him is lost. Not one word does he address to his wife, not one inquiry does he make respecting his child, from the time of deserting his own home to the time of his return to England. It is not known to his family or his friends during all this weary period whether he is living or dead. But the reader, who first meets this interesting character on board the *Argus*, in the chops of the Channel, is required to accept him as an individual overflowing with all those pleasant longings, hopes, and aspirations which are presumed to be proper to the least blameworthy wanderer returning homewards; and possessed of so acute an affection for his deserted wife and child, that the loss of reason or life is to be dreaded if death should, in his absence, have removed the one or the other. Nay, more; so great is the affection, so nobly sensitive the feelings he displays, that, contrasted with them, the acts and emotions of the husband who has sacredly performed at all times and under all circumstances, his duties to wife and children, become commonplace! The deep-seated, irrepressible, and inexhaustible affection of George Talboys—the man who, by the cruel desertion of his wife, gave rise to her subsequent fantastic criminality—is the clear mirror, so to speak, in which is reflected the dark shadow of Lady Audley's, otherwise Mrs. Talboys', failings. This apotheosis of pseudo-sentiment has an important bearing upon the subject-matter of this article, as will be seen in the sequel.

George Talboys and his friend Robert Audley visit Audley. It is nightfall; a storm is gathering; and Talboys is exceedingly discomposed, having seen Lady Audley's portrait in her boudoir, and subsequently met her ladyship under the archway leading to the Court, as she is returning home in a fly. Robert Audley is puzzled with his friend's irritability and moodiness. The storm has broken, and Talboys seeks, but unavailingly, to cool his mysterious excitement in the pouring rain.—"There was nothing for it but to leave Mr. Talboys to himself, to recover his temper as best he might. 'He was irritated at my noticing his terror at the lightning,' thought Robert, as he calmly retired to rest, serenely indifferent to the thunder, which seemed to shake him in his bed, and the *lightning playing fitfully round the razors in his open dressing-case.*" (L. A. S. i. 149.)

George Talboys is missing from Audley. Robert Audley supposes that his friend has returned to London, but he is, nevertheless, uneasy.—"Why should he be so?" asks Lady Audley, as she gossips with Mr. Robert at the tea-table in the drawing-room of Audley Court, present also Sir Richard and Miss Audley:—

"'I'll tell you why, Lady Audley,' answered the young barrister. 'George had a bitter blow a year ago in the death of his wife. He has never got over that trouble. He takes life pretty quietly—almost as quietly as I do—but he often talks strangely, and I sometimes think that one day this grief will get the better of him, and he'll do something rash.' Mr. Robert Audley spoke vaguely; but all three of his listeners knew that the something rash to which he alluded was that one deed for which there is no repentance." (L. A. S. i. 172.)

Mr. Maldon, Lady Audley's unacknowledged father, resides in Brigsome's-terrace, Southampton.—"Brigsome's-terrace was perhaps one of the most dismal blocks of buildings that was ever composed of brick and mortar since the first mason plied his trowel and the first architect drew his plan. The builder who had speculated in the ten dreary eight-roomed prison houses had hung himself behind the parlour-door of an adjacent tavern while the carcases were yet unfinished." (L. A. S. ii. 16.)

Lady Audley is in her dressing-room the morning after she had set fire to the public-house.—"Her pale face seemed to grow paler as the morning advanced. A tiny medicine-chest was upon the dressing-table, and little stoppered bottles of red lavender, sal-volatile, chloroform, chlorodyne, and ether, were scattered about. Once my lady paused before the medicine-chest, and took out the remaining bottles, half absently perhaps, until she came to one which was filled with a thick dark liquid, and labelled, 'Opium—Poison.' She trifled a long time with this last bottle, holding it up to the light, and even removing the stopper and smelling the sickly liquid. But she put it from her suddenly with a shudder. 'If I could!' she muttered, 'if I could only do it! And yet, why should I *now?*' (L. A. S. iii. 54.)

Again, when, by a daring feat of literary gymnastics, Lady Audley is imprisoned in a Belgian asylum, she exclaims:—"I would kill myself, and defy you, if I dared; but I am a poor, pitiful coward, and have been so from the first." (L. A. S. iii. 169.)

The story hastens to an end. George Talboys is restored to his family, and Robert Audley seeks to ingratiate himself with Miss Talboys.—"How pleasant it was to be lectured by the woman he loved! How pleasant to humiliate and depreciate himself before her! How delightful it was to get such splendid opportunities of hinting that, if his life had been sanctified by an object, he might, indeed, have striven to be something better than an idle *flaneur* upon the smooth pathways that have no particular goal; that, blessed by the ties which would have given a solemn purpose to every hour of his existence, he might indeed have fought the battle earnestly and unflinchingly. He generally wound up with a gloomy insinuation to the effect that it was only likely he would drop quietly over the edge of the Temple Gardens some afternoon, when the river was bright and placid in the low sunlight, and the little children had gone home to their tea. 'Do you think I can read French novels, and smoke mild Turkish, until I am threescore-and-ten, Miss Talboys?' he asked. 'Do you think that there will not come a day in which my meerschaums will be foul, and the French novels more than usually stupid, and life altogether such a dismal monotony, that I shall want to get rid of it somehow or other?'" (L. A. S. iii. 260.)

John Mellish, the genial and sanguine Yorkshireman, the second husband of Aurora Floyd, is described as a man who,—"saw all things in the universe as he wished to see them; all men and women good and honest; life one long, pleasant voyage in a well-fitted ship, with only first-class passengers on board. He was one of those men who are not likely to cut their throats, or take prussic acid, upon the day they first encounter the black visage of care." (A. F. ii. 6.)

Conyers, the training-groom, and first husband of Aurora Floyd, erroneously supposed by her to have been accidentally killed on the Continent, enters the service of Mr. Mellish. Mrs. Mellish, by a heavy bribe, seeks to secure Conyers' silence, and to induce him to leave Mellish Park. She appeals to her father for the large sum of money Conyers requires:—"'Father! father!' she cried, with a sudden change of her voice and manner, 'I am hemmed in on every side by difficulty and danger; and there is only one way of escape—except death. Unless I take that one way, I must die. I am very young—too young and happy, perhaps, to die willingly—give me the means of escape.'" (A. F. ii. 153.)

Conyers is shaving himself after a night's dissipation,—"His eyes were dim and glassy: his tongue hot and furred, and uncomfortably large for his parched mouth: his hand so shaky that the operation which he performed with a razor before his looking-glass was a toss-up between suicide and shaving." (A. F. ii. 127.)

Conyers, seeking an outlet for the irritability engendered by the night's dissipation, vents it on his attendant, Steeve Hargrave, the "softy."—"There is a great deal of vicarious penance done in this world. Lady's-maids are apt to suffer for the follies of their mistresses. Lady Clara Vere de Vere's French abigail, is extremely likely to atone for young Laurence's death by patient endurance of my lady's ill-temper, and much unpicking and remaking of bodices, which would have fitted her ladyship well enough in any other state of mind than the remorseful misery which is engendered of an evil conscience. *The ugly gash across young Laurence's throat,* to say nothing of the cruel slanders circulated after the inquest, may make life unendurable to the poor, weak nursery-governess who educates Lady Clara's youngest sisters; and the younger sisters themselves, and mamma and papa, and my lady's youthful confidantes, and even her haughtiest adorers, all have their share in the expiation of her ladyship's wickedness." (A. F. ii. 210.)

Mrs. Mellish looks into a mirror, as, under the terror of Conyers' murder and its consequences, she is about to fly from her husband's house.—"The face which she saw in the glass was very pale and rigid; the large, dark eyes dry and lustrous, the lips drawn tightly down over the white teeth. 'I look like a woman who would cut her throat in such a crisis as this,' she thought; 'how often I have wondered at the desperate deeds done by women! I shall never wonder again.'" (A. F. iii. 45.)

Suicide is an act neither unfamiliar in England, nor ignored by English writers of fiction. But for a parallel to the fashion in which self-murder is dealt with in *Lady Audley's Secret* and *Aurora Floyd,* recourse must be had to the most debased works of French novelists. With the English writers of the present day, as a rule, suicide is the tragic culmination of utter misery or of criminal folly; the act of an insensate, or of those whose sense of moral accountability has been blunted or extinguished by wretchedness or immorality; a crime against the State, a sin against God. If it has been sought to enlist compassion for the unhappy suicide, sympathy has never been asked for the crime itself; nor has it been attempted to deck the latter with spurious sentiment. At the most, where the ghastly fact has not at all times been treated with that gravity which befits so tragic a subject, an absurd colloquialism may have been indulged in, such as men under the influence of transitory vexation or from a mere redundancy of vigour and satiety of prosperity, may occasionally give expression to. But in *Lady Audley's Secret* and *Aurora Floyd* suicide is dealt with far otherwise. There the idea is intruded into the brightest as the darkest aspects of the stories; it is suggested in their pleasantest as well as their most miserable scenes; it serves as the foil to happiness, the legitimate accompaniment of wretchedness, folly, or wickedness, and it is made to give sharp point to a playful simile.

John Mellish, the genial, honest-hearted, simple-minded, jovial, and most pleasing Yorkshireman, suggests—SUICIDE. Robert Audley, happy in having freed his uncle from an infamous alliance; happy in the restoration of a much-missed friend; more happy in a reciprocated attachment, suggests—SUICIDE. George Talboys, shrinking from the results of his own thoughtlessness, and the reproaches of his wife, suggests—SUICIDE. The flickering lightning of a passing storm, after nightfal, as it plays over the polished fittings of a dressing-case, suggests—SUICIDE (or *murder*). George Talboys first missing from Audley, suggests—SUICIDE. A row of dismal, unfinished houses, suggests—SUICIDE. Lady Audley in her dressing-room waiting for tidings of the arson she had committed, suggests—SUICIDE. Lady Audley housed in the Belgian asylum, suggests—SUICIDE. Aurora Floyd driven almost to despair by the return of Conyers, suggests—SUICIDE. Aurora Floyd, in despair, about to fly from Mellish Park, suggests—SUICIDE. Conyers shaving himself with trembling hand suggests—SUICIDE. Conyers venting his spleen on Steeve Hargraves, suggests—SUICIDE.

The frequent recurrence of the idea of suicide would alone rivet attention, apart from the incongruity of the scenes giving rise to it. Suicide, as a fact, does not form any portion of the machinery of either of the two stories, but the notion is rarely absent from the reader's mind. Self-murder, or murder, is, indeed, being almost continually suggested by the authoress, giving singular ghastliness to both novels. It is of interest in connexion with this strange harping upon suicide, to note the manner in which murder is referred to. Three examples will suffice for this purpose.

Phœbe Marks is speaking to Lady Audley of Luke, her betrothed.—"I daren't refuse to marry him," she says. "I've often watched and watched him, as he sat slicing away at a hedge-stake with his great clasp-knife, till I have thought that it is just such men as he who have decoyed their sweethearts into lonely places, and murdered them for being false to their word." (L. A. S. i. 219.)

Conyers is in affable gossip with Steeve Hargraves:—"'Well, I don't think you are over-valiant,' answered Mr. Conyers, 'to be afraid of a woman, though she was the veriest devil that ever played fast and loose with man.'—'Shall I tell you what it is I'm afraid of?' said Steeve Hargraves, hissing the words through his closed teeth in the unpleasant whisper peculiar to him. 'It isn't Mrs. Mellish—it's myself. It's *this*—' he grasped something in the loose pocket of his trousers, as he spoke, 'it's *this*:—I'm afraid to trust myself anigh her, for fear I should spring upon her, and cut her throat from ear to ear. I've seen her in my dreams, sometimes, with her beautiful white throat laid open, and streaming oceans of blood; but for all that, she's always had the broken whip in her hand, and she's always laughed at me. I've had many a dream about her; but I've never seen her dead or quiet; and I've never seen her without the whip.'" (A. F. ii. 79.)

The final interview between Aurora Floyd and Conyers:—"Aurora's passion had reached that point in which all consciousness of external things passes away in the complete egoism of anger and hate. She did not see his superciliously indifferent look; her dilated eyes stared right before her, into the dark recess from which Captain Prodder watched his sister's only child. Her restless hands rent the fragile border of her shawl in a strong agony of her passion. Have you ever seen this kind of woman in a passion? Impulsive, nervous, sensitive, sanguine; with such a one passion is a madness—brief, thank Heaven! and expending itself in sharply cruel words, and convulsive rendings of lace and ribbon, or coroner's juries might have to sit even oftener than they do. [*Murder or suicide, or both?*] It is fortunate for mankind that speaking daggers is often quite as great a satisfaction to us as using them, and that we can threaten very cruel things without meaning to carry them out." (A. F. ii. 264.)

There is a rare power of expression and observation in this passage, as in many other passages, of *Aurora Floyd*. Would that the possessor of these great gifts had given to them a more noble development! But the future is still before her.

The peculiar disposition to dwell upon the ideas of murder and suicide which is so conspicuous in *Lady Audley's Secret* and *Aurora Floyd*, may probably be traced to a single source. It is only in popular French literature that an approximation can be found, at the present day, to that frequent recurrence to the idea of suicide which is so curious a characteristic of these novels. Certainly the fact of suicide plays no inconsiderable part in the current news of this country; but both our periodical and dramatic literature are entirely free from that objectionable tendency to play with the idea of self-murder, and deck out the act in meretricious colours, which is observed on the stage and in the daily journals of France. Moreover, there is a class of fiction across the Straits, neither the least admired nor the least ably written—even if in one sense the most debased, as it undoubtedly is the most baneful of the many productions which issue from the teeming press of our neighbours—in which suicide becomes a kind of fetich. The works of this class are chiefly distinguished by the pseudo-scientific scepticism which pervades them; by the elevation of sensuality to the dignity of the highest virtue, and the degradation of continence to the station of a pitiful vice; by the tricking out of immorality in a garb of religious phraseology, and by the uplifting of suicide as a legitimate panacea against all ills. These works, in fact, belong to the *Werther* school of literature, which under the immediate influence of its great prototype had but a short-lived existence in England at the termination of last and beginning of this century, but which has flourished in France from the period of publication of the inimitable, but alas! most injurious *Sorrows*, to the present time, and still flourishes there with, if anything, increasing vigour. The peculiar characteristics of this school of literature—or, as it may be conveniently designated, of *Wertherism*—have been examined in detail at a recent date in the pages of this Journal, and it is sufficient for the present to have recalled them to memory.*

Five years ago we suggested a doubt, and three years ago raised the question, whether there was about to be a recrudescence of Wertherism in England. The avidity with which Mr. Buckle's crude *quasi*-scientific speculations on suicide had been received—speculations which tended to set aside freedom of choice and individual responsibility in the production of the act;—the strange fascination exercised by the opera of *La Traviata*, and the utter indifference of the public to the abominable nature of the story on which it was founded—a story accurately reproduced in the libretto, of French origin, and which, thanks to the flattering reception of the opera, is now proffered for sale in an English guise, at a trifling cost, to ingenuous youth of both sexes, together with other works of the same class;—finally, the probability that suicide was about to become a favourite subject with our artists—self-murder having assumed a somewhat prominent position upon the walls of the Academy in 1859 and 1860:—these things at least indicated a predisposition to Wertherism, if they were not the forerunners of, or did not determine, an outbreak. But be this as it may, there can be no doubt that England at the present moment is suffering from a recrudescence of Wertherism. It is no part of our purpose to attempt to show to what extent, or in what manner, the circumstances we have noted may have fostered or determined this outbreak. We would briefly indicate the facts which prove that Wertherism constitutes the essential characteristic of a form of literature which has recently undergone a singularly rapid development, and become very rife, among us.

The class of works which, approaching this subject from a peculiar point of view, we have designated as belonging to the Werther school of literature, is more familiarly known as the *Sensation Novel*. A morbid exaggeration of feeling is peculiar

---

* See the articles on the *Classic Land of Suicide*, vol. i. p. 182, and the *Æsthetics of Suicide*, vol. i. p. 563.

to all the works of this class of fiction, and, in the majority of them, sentiment is upheld as the proper criterion to which the acts and doings of the characters of the story are to be referred for judgment—sentiment, in fact, usurps the province of judgment. This is observable in the character of George Talboys, in *Lady Audley's Secret*, as already noted. In several of these works the interest chiefly turns upon the ties of marriage being represented in an obnoxious light, as darkening, for example, the otherwise pleasant course of high-souled bigamy and adultery. In others an apology, implied or expressed, is offered for adultery and clandestine prostitution, and the wretched paradox is maintained of sensual vice being a disguised virtue.

"I consider the ceremony of marriage as one of the most absurd inventions ever inflicted on human beings by mortal man," the heroine of one of the best known of these works is represented as saying. . . . "In the first place, do we not swear to *love* always and to the end, when to do so is too often clearly and simply out of our power? Is human love the growth of human will? Certainly not; and as certainly is it only as words of course, that we vow 'to honour and obey' the man who may turn out a dishonourable wretch, or a monster of tyranny and oppression." This fair lady, consistently with her principles, is depicted as becoming the mistress of the man she loves. In the ordinary progress of affairs he is married to another lady, and she is cast aside. A pattern of wrongfully-used virtue, she, for a time, leads a life of poverty. But presently the lawful wife of her former lover becomes unfaithful, and the distressed husband seeks consolation in the unabated affection of his late mistress. In the end the latter becomes the legatee of his whole property, and presents an example of wealthy virtue, equalled only in its high-mindedness by the virtue she had displayed when in poverty, and putting to shame the common plodding virtue of honest, everyday life.

In another of these works, written by an author of rare ability, and which is chiefly intended as a protest against the cruel operation of the law which does not permit children born out of wedlock to be made legitimate by the subsequent marriage of their parents, we read (the family solicitor being presumed to be speaking):—"I am far from defending the law of England, as it affects illegitimate offspring. On the contrary, I think it is a disgrace to the nation. It visits the sins of the parents on the children; it encourages vice, by depriving fathers and mothers of the strongest of all motives for making the atonement of marriage; and it claims to produce these two abominable results in the names of morality and religion. But it has no extraordinary oppression to answer for, in the case of these unhappy girls [children of a second wife born during the lifetime of the first, unable to inherit on account of illegitimacy, and left penniless from the father having made no provision for them, an untimely death preventing his intentions in this respect being carried into effect]. The more merciful and Christian law of other countries, which allows the marriage of the parents to make the children legitimate, has no mercy on these children. The *accident* of their father and mother having been married when he first met with the mother has made them outcasts of the whole social community; it has placed them out of the pale of the Civil Law of Europe." Truly morality must be at a heavy discount when it can be suggested as a preliminary step to a modification of the law which determines the social position of illegitimate children, that marriage should be regarded as an *accident!*

In a third work of fiction of this class, the author derives his inspiration from Shelley's religious scepticism and loose notions on marriage. The hero has early become attached to a young girl, but having promised his mother on her death-bed not to marry until he is twenty-one years of age, he compromises the matter between his feelings and his promise by making the girl his mistress. Meanwhile, his relatives, anxious for his welfare, have sought out a lady suited to be his wife when his fifth lustrum shall have ended. His mistress learning this, conceives the idea that she may prove an impediment to her lover's prosperity. Thereupon she determines to sacrifice herself, in order to promote his welfare. First, she endeavours to alienate his affections by making an assignation with another man and appearing with him in public at a representation of *La Traviata;* and then she poisons herself by taking strychnine. Twelve months after this event the hero marries the lady selected by his family, and on the wedding-night blows out his brains in a melodramatic fashion. The incidents of this story require to be interpreted by the light of "that *higher faith,*" to adopt an expression of the author's, "which may exist with honest doubt, or even a bold denial of that puerile conception, the God of the Priests."

Other examples might be cited, if it were needed, but these will suffice to show the recent vigorous outgrowth of Wertherism in English literature. Brief though our illustrations have been, we can clearly recognise in them the great characteristics of the Werther school of fiction—the morbid exaggeration of feeling; the scepticism by which man panders to his sensuality; the tendency to confound vice and virtue, and unsettle the fundamental notions of morality; and the habit of invoking suicide as a legitimate means of escape from evils of our own creation. Add the babble of suicide which is found in *Lady Audley's*

*Secret* and *Aurora Floyd*, and the parallel is complete according to the latest French examples of the same school of fiction.

It is worthy of note, in connexion with the suggestion that the two last-named novels, as doubtless their congeners, have derived their inspiration from a French source, that in the former of these there are several curiously significant allusions to French fiction. Phœbe, Lady Audley's maid, "knew enough of the French language to dip into the yellow-paper-covered novels which my lady ordered from the Burlington-arcade, and to discourse with her mistress upon the questionable subjects of those romances." (L. A. S. i. 212.) Robert Audley returns to town, after his interview with George Talboys' father and sister:—"The snug rooms in Fig-tree-court seemed dreary in their orderly quiet to Robert Audley upon this particular evening. He had no inclination for his French novels, though there was a packet of uncut romances, comic and sentimental, ordered a month before, waiting his pleasure upon one of the tables." (L. A. S. ii. 111.) Again: "If Robert Audley had lived in the time of Thomas à Kempis, he would very likely have built himself a narrow hermitage amid some forest loneliness, and spent his life in tranquil imitation of the reputed author of the *Imitation*. As it was, Fig-tree-court was a pleasant hermitage in its way, and for breviaries and Books of Hours, I am almost ashamed to say the young barrister substituted Paul de Kock and Dumas *fils*." (L. A. S. ii. 122.) In a previous quotation we have already seen how a playful allusion to suicide is associated with a reference to the young barrister's predilection for French novels.

As a further illustration of the degraded sentiment for which the sensation novelist is apt to solicit the sympathy of readers, the character of Miss Talboys in *Lady Audley's Secret* is worthy of passing notice. Miss Talboys is the high-souled lady of the story, the representative of female virtue and dignity. She has learned from Robert Audley his belief that her brother has been murdered, and his suspicions of the murderess. She exclaims:—" 'I have had no one but my brother. All the love that my heart can hold has been centred upon him. Do you wonder, then, that when I hear that this young life has been ended by the hand of treachery, that I wish to see vengeance done upon the traitor? Oh, my God!' she cried, suddenly clasping her hands, and looking up at the cold winter sky, 'lead me to the murderer of my brother, and let mine be the hand to avenge his untimely death!' Robert Audley stood looking at her with awe-stricken admiration. Her beauty was elevated to sublimity by the intensity of her suppressed passion . . . . . 'Miss Talboys,' said Robert, after a pause, 'your brother shall not be unavenged.' (ii. 94) . . . 'We may both be deceived; your brother may still live.' 'Oh! if it were so,' she murmured, passionately, —'if it could be so.' 'Let us try to hope that it may be so.' 'No,' she answered, looking at him through her tears, 'let us hope for nothing but revenge.'" (ii. 98.)

Not a trace of pity for the woman who had been infamously deserted by her brother; not a word of justice. Revenge alone occupies the thoughts of this tearful woman; and the most degraded of all human passions, which, when actively displayed, commonly gives to the countenance a diabolical expression, fills her aspect with sublimity! She would avenge her brother's death with her own hands. If the most virtuous female characters of the sensational school may be righteously impelled to murder, truly self-murder becomes a very venial offence, if offence at all. But, alas! that the noblest female character in *Lady Audley's Secret* should be closely linked to the most degraded male character in *Aurora Floyd.* Yet so it is. If our worst passions are to be elevated to the rank of virtues, or *quasi*-virtues, surely those who possess them in their fullest vigour should be elevated also in the moral scale. Conyers, the infamous groom, is talking with Mrs. Mellish:—" 'You understand—two thousand down. That's *my* alternative; or I leave the place to-morrow morning —with all belonging to me.' 'By which course you would get nothing,' said Mrs. John Mellish, quietly. 'Shouldn't I? What does the chap in the play get for his trouble, when the blackamoor smothers his wife? I should get nothing—but my revenge upon a tiger-cat, whose claws have left a mark upon me that I shall carry to my grave!'" (A. F. ii. 112.)

It may be said that, even admitting the existence of the essential characteristics of Wertherism in our sensation novels, the evil at least is neither so glaring nor so obnoxious as in France. If certain of our writers of fiction are indulging in scepticism, it is not the ultra-scepticism met with in French novels; if at times they adopt the phraseology of morality to shroud certain forms of immorality, they do not prostitute the most sacred ideas and expressions to the service of overt sensuality; if they have sought to modify prevalent notions on the iniquity of adultery and prostitution, they have not pleaded for the righteousness of the former sin, nor granted an apotheosis to the latter; if they have tampered with suicide, they have not as yet become the open apologists of the act. All this may be true, but the evil is not the less grave because it is less glaring in form. Nay, with us, the gravity of the evil is to be measured by the insidiousness of its operation rather than by its patency. It is improbable that public feeling in this country will ever attain a degree of insensitiveness such as to admit of that utter negation of morality which distinguishes French fiction of the Werther school; but it is, nevertheless, most certain that recent writers have sought to engraft the principles of this school on English literature, and it is well that we should mark to what these principles may lead, and what are the patterns held up for imitation.

It is somewhat curious that the latest French apologist for suicide, as the latest English novelist who has coquetted with self-murder, is a lady. Her work is deserving of attention, not only as a recent example of the æsthetical literature of suicide, but also as an illustration of the class of fiction which furnishes the model for our "sensation novels."

*Gaëte* is the title of this story, and, with that pseudo-scientific pretentiousness which is peculiar to this class of fiction, it is further styled "a psychological study." Madame Maria de Fos is the authoress. The heroine is the daughter of a French viscount, motherless, and utterly neglected by her father, who is a gambler and a villain. A worthy priest watches over her childhood, secures her an education in a religious establishment, and, when she reaches womanhood, places her as a dependent in the house of the Baroness of Velaco, who receives her as a companion. Gaëte is surpassingly beautiful, amiable, intellectual, and accomplished. The baroness has an only son, who is an epileptic imbecile. Gaëte quickly secures the affection of her protectress, who treats her as a daughter, makes a handsome provision for her future livelihood, and saves her father from being imprisoned for debt, but who, nevertheless, does not hesitate to sacrifice her unhappy companion in order to promote her son's welfare. Acting upon Gaëte's exaggerated sense of gratitude for the kindness and protection which had been extended to her, she induces her to marry her son. The hideous match is completed, and Gaëte becomes the wife of the young baron of Velaco. The marriage causes but little change in the relations between herself and the dowager baroness. "From time to time, it is true," the story runs, "a look was exchanged between them, in which this affectionate mother seemed to express a feeling of compassion, and to demand absolution from her victim, which the young wife accorded without reserve. She had paid her debt, and was free from all obligation. As to Josias [the baron] he continued childishly indifferent, a melomaniac, and did not appear to comprehend the sacrifice which had been made. Gaëte had subjected herself to a great trial, and when she was about to become a mother, she felt that, if God permitted her children to live, they would certainly require greater tenderness and affection than others; but she resolved to do her duty, whatever the cost might be. 'Has not God himself,' she thought, 'carried His cross even to the summit of Golgotha?'"

We cite this passage to show in what manner that "*higher faith*," as it is designated by one of the English writers to whom we have already referred, and which is affected by the authors of this form of fiction, deals with the most sacred subjects.

Years pass on, the dowager baroness is dead, and Gaëte is the mother of three children, all sickly, and tainted with the sad infirmity of their father. Troubles accumulate around the unhappy wife; the mental faculties of the husband become more and more clouded; while, to fill up her measure of suffering, the very means of subsistence are taken away, and the ancient mansion and estate of her husband's family alienated, by the rascality of her father. She now takes a desperate resolve. Securing what little ready money she can gather together, she leaves her husband and children in charge of an old nurse, and hastens secretly to a German spa. There she seeks to place her money to good account at a gaming-table, but fortune fails her, and she loses all. Then she unhesitatingly becomes the mistress of a wealthy nobleman, and strives to lay aside, from the earnings of her prostitution, sufficient to maintain her ailing children and rapidly decaying husband in comfort for the brief remainder of their lives.

Picture our heroine at midnight in the splendid villa of her wealthy gallant, where the rooms are flooded with light and crowded with all the well-dressed, disreputable loungers at the spa. "In a more worthy medium," says the story, "she would have been taken for a chaste duchess; but in that where she was found, she seemed to be the shade of virtue, forced by destiny to protest, by her presence, against cynicism and unchastity." (This is the æsthetical aspect of her revolting prostitution.) The rattle of the dice, and the monotonous clamour of the gamesters, as they bet upon the cards "in the sacramental terms moulded in the slang of hells and introduced even into decent drawing-rooms," echo around the tables, while in a room apart sits Gaëte, a group of the more select guests around her. Neither encouraging nor avoiding conversation, she accepts with weary indifference the "banal courtesy" of her companions. Presently a player "jocosely mentions the name of an Englishman who had killed himself in the morning after a heavy loss at cards, and whose suicide had made so much noise among the bathers, that the local authorities and the managers of the gaming-houses had, by a common and immoral accord, sought by every means to hide it."

"On hearing this story, the physiognomy of Gaëte was suddenly illuminated with a ray of life and intelligence. She shook her head, as if to chase away heaviness and fatigue, and with a calm voice, such as profound conviction or long acquaintance with the world alone gives, she commenced, to the astonishment of all who heard her, the apology of suicide:—

"How is this, gentlemen, that you dare to blame the memory of Lord X—— because he has thought proper to shuffle off the world, where, doubtless, suffering and deception were his sole lot? Do you know the causes which have prompted him to commit an act which is never accomplished, whatever is said to the contrary, without having been long and maturely considered? What are the circumstances which, most probably, led to his resolve? Insanity is out of the question: it consists, physiologists tell us, in complete derangement of the mental faculties, and the man who is thus deprived of his freewill is but an agglomeration of matter, distinguished from the plant by movement only, and too closely allied to the least noble animals by certain physical wants. Whether this state be permanent or transitory, whether it be occasioned by excess of tension of the fibres which compose, or by a physical accident, such as compression of, the brain, the man who is afflicted in this manner can no longer be reckoned among the creatures whom God has endowed with a superior essence, and no moral law can be applied to him. But ought this exemption to be extended to the individual who, exceptionally and suddenly excited by a sensation exceeding his intellectual force, has sought rest elsewhere? Is it necessary to dishonour the unfortunates who, enfeebled by long suffering, have been led by philosophical reflections to commit suicide? You cannot think so, and let the opinion be what it may which influences you most at the present moment, no one would wish to pronounce a judgment which concerns that which man holds most precious, that is to say his reason and his life.

"I have often heard it said that courage is not wanting to support the pains of existence; but what are those pains? What is this existence? Should we consider as genuine torments those which society has itself created by imposing obligations which most frequently it is impossible for man to fulfil. Ought the members of this society, who, unknown to themselves, and without their consent, have been united in the great social body, to accept these obligations? Yes, you will answer me. I am of the same opinion in so far as it relates to those beings with whom we are allied by blood. The great natural and primordial laws overrule all individual considerations, and every one who revolts against them ought to be put down. But does the same rule hold when all solidarity has disappeared? Every individual has his part traced in the providential pandemonium. Since to each one relative forces have been assigned, if in consequence of a human aberration, a too heavy burden should rest upon a member of the association, he ought to have the power to disembarrass himself of it, if he would not have the weight fall upon another more feeble still.

"We may dispute the right of a man of superabundant capacity, placed at the head of other men to enlighten and conduct them, disposing of a life which appertains to all. We may contest the freedom of a father of a family to die, he having voluntarily constituted himself the guardian of feeble beings who are fragments of himself. Wherever social and natural laws are mingled together, becoming *united*, there is harmony, and both concur in forcing a father or a mother to maintain, rather than abandon, the post of honour which God has given to them, and which society has confirmed. But if it be said that we are forbidden by religious laws, and it is pretended that our lives do not belong to ourselves, the obligation being thus forced upon us of suffering the most frightful torments with the sole object of being agreeable to God who placed us in the world,—becoming a prey to unhappiness, as it is maintained, in prospect of a future recompense; then my reason ceases to agree with a stupid belief, and looking around me, collecting in the scales of a balance, as it were, the *for* and *against* of the causes of my being, if an equilibrium is not established—if ennuis, chagrins, privations, miseries, and shame overweigh natural obligations and social duties, I believe that I am free to act without taking into account the prescriptions of an impossible morality.

"If suicide was a crime of lèse-nature, it would never be committed by a being living in a savage state. But what do we see at every step in those regions where civilization has not penetrated?—men who have become old, regarding themselves as useless and a charge to their children, not hesitating to commit suicide in order to put an end to their evils and remove the burden their existence imposes. Is the gardener guilty of a crime who pitilessly separates from the trunk the feeble or parasitic branch which is prejudicial to its development? Why, then, should we blame the individual who thinks fit not to await his condemnation, but who proceeds to remove himself when it becomes necessary?

"To act in this way seems to me more worthy than to believe ourselves indispensable to others. Can it be, gentlemen," said Gaëte, becoming more and more animated, "that any one of you is so useful to society that his loss would prove an impediment to the progress of the great social and humanitarian order which Providence has designed? Let me tell you, apart from the esteem which I entertain for each of you individually, that it seems to me little disturbance would arise from the disappearance of one or of many of you; and I boldly avow that if I heard to-morrow that one or the other of you had thought fit to terminate his existence because he had overstepped, in the stakes he has played this evening, the limits which his fortune and his honour ordained him to respect, I should not pity him, but, on the contrary, I should approve the act. He would have adopted the only course left to him by society and morality. If society has not furnished other means of escape, so much the worse for it. Until society has settled this question by providing means, it has no cause of complaint.

"Some of you have blamed this Englishman whose body was found in the lake, which may be perceived from here, sombre and tranquil, notwithstanding the drama ended in its waters; others have had pity; with the majority the event has been regarded with indifference, and to-morrow it will have been completely forgotten by the visitors, and *Rouge et Noir* will not the less continue and swallow up your fortunes.

"This suicide, this crime, as it pleases the world to term it, will prevent no one submitting to chance the things in which the joys and griefs of the world are made to consist. Is not this the best proof that the sentiment is not in agreement with the opinion, I do not say of the public, but publicly professed? Shame arrests no one; for there is no shame where energy is required for the accomplishment of an act—unless from this act, as I have already said, springs the sufferings of persons shamefully abandoned.

"If suicide was considered as a crime by the legislator, the Diet of Frankfort would long ago have compelled the petty princes of the Confederation to abolish those infamous gambling-houses which continually give rise to it. But as it is, these houses exist under the protection of the law—under the safeguard of the police, who receive even a portion of their profits—befouled though they be with the blood of suicides and the ruin of family honour. What do I say? under the protection!—nay, these houses are shielded by the tenderness and affection of the ruling powers, as may be most easily demonstrated.

"You have witnessed the efforts made by the authorities to hide the event of yesterday. Observe, now, what becomes of the unfortunates whom they have tempted, by every means of publicity, to come here and ruin themselves. Some, when the rags in which they are dressed would have cast a sad shadow upon the golden wainscots, or when want has forced them to seek alms, have been forbidden to enter the saloons. Others, on the pretext of a wretched debt contracted to avoid dying from hunger, have been given an asylum in prison. To a few, perhaps, the smallest number, a few pieces of silver have been cast to facilitate their departure, but it was by the hands of a police-agent, commissioned to conduct them over the frontier, that the succour was given. There they have been abandoned, with the designation "*a ruined gambler*" affixed to them, so that society may always know them, and refuse them bread.

"And you, gentlemen, would forbid suicide to these wretches! You would contest their right to dispose of a life for ever lost! You would make them criminals after having made them miserable! Truly, you are in a dilemma from which there is no escape. Sooner or later you will admit, with me, that there are situations in which it is needful to die—where death is a right, a duty—a force of which nothing can divest us, until the time when great social reforms shall have been consummated."

Such is Madame de Fos's apology for suicide. The similarity existing between the "philosophical reflections" which, according to her reasoning, determine the act, and Mr. Buckle's speculations on the same subject, first fix the attention. "Society," Madame de Fos maintains, approaching the subject from a transcendental point of view, "imposes obligations which, most frequently, it is impossible for man to fulfil: if these obligations weigh unbearably upon the individual, and he rids himself, by death, of the burden, he is not to be held accountable." Mr. Buckle, approaching the subject presumably from a statistical point of view, but, in reality, attempting to bolster up by mangled, nay worse, misrepresented figures, a figment of imagination, engendered in the same school as that in which Madame de Fos has studied, asserts that all evidence points—"to one great conclusion, and can leave no doubt on our minds that suicide is merely the product of the general condition of society, and that the individual felon only carries into effect what is a necessary consequence of preceding circumstances."* The *Bhagavad-Ghita* instructs us that—"The presumptuous thinks himself the author of his actions; but all his actions come from the force and from the necessary concatenation of things." Cousin tells us that this dogma is "destructive of all liberty and all morality:"† but Mr. Buckle's and Madame de Fos's speculations on suicide are simply a modern development of it; and the "apology" of the latter furnishes conclusive evidence of its destructiveness to all morality. The world in Madame de Fos's fiction becomes a "providential pandemonium;" God a name, useful at times as a pivot for fanciful phraseology, but nothing more; religion a stupid belief, or at best a scheme of impossible morality; morality a matter of individual taste and convenience; vice—and if vice why not crime?‡—a product of the general condition of society, for which the individual man is not responsible; while society or general humanity is treated and spoken of as a species of demon. "The two men who knew best the mystery of Gaëte's life," says our story, in respect of her prostitution, "the sole act which she could not avow publicly, and after which she had sworn not to live, not only did not contemn her, but they acknowledged at her feet [by offering their hands in marriage] that there is nothing absolute in human considerations; that immutability belongs but to God, and that it is an error to apply the term to the things of earth. If, in truth, Gaëte had committed a fault, in ceding one day to a pressure which exceeded the limits of female power, the causes which had led to an act of despair were sufficient to remove this act from the animadversion of the world. It should suffice for the re-establishment of her good fame, and in order that she should come triumphant from the bar of public opinion, that two great hearts [each, by the way, had resolved to kill the

* *History of Civilization*, vol. i. Introductory chapter.

† *Cours de l'Histoire de la Philosophie Moderne.* 6$^{me}$ Leçon.

‡ See Mr. Buckle on the statistics of *Murder*. Op. cit. Introductory chapter.

other, or be killed, if Gaëte had accepted either one as husband] had become her judges. Let all human actions be submitted to a like proof, and if society does not modify its judgments, it will be because it has misunderstood the most holy of all laws, that which emanates from the conscience, which is God!"

The negation of morality and religion is complete; but there is still a lower depth of blasphemous perversity.

Gaëte returns to her home; her husband, and two of her children, are dead, the third is dying, and she waits but its death to commit suicide. The venerable priest who watched over her childhood seeks to turn her from her fatal purpose. "But honest and conscientious above all things, he claims not to impose upon her his belief. If he himself had received nothing from the world, neither had he lost anything. In presence of a female, weighed down by grief, deprived of all hope, devoted to solitude and sorrow, the old man knew not what to say, because he dare not oppose more obstinately than he had done the most sacred of all rights, that of disposing of one's-self—that of liberty. . . . . His last word was a benediction, and at the same time a full absolution—'Whatever thou decidest, O my daughter,' he said, as he was about to depart, 'thy sin is already expiated. God has inspired thy resolutions. My duty was to sustain thee, not to blame, because thou wishest not consolation on earth.'"

Truly the subversion of all sound principle could not be more complete! Right and wrong are meaningless terms; vice is confounded with virtue; sin is heaven-born; suicide a portal to bliss; and, to complete all, the Church is called in to bless this annihilation of Christian doctrines and practice!

And yet English literature has of late become appreciably infected with these infamous teachings. Their influence is chiefly exercised upon young people; and the great excess of suicides among the youthful in Paris is one of the surest indications of their baneful effects. The most formidable obstacle to the further extension, in this country, of a class of fiction which has derived its principal interest from the greater or less extent to which it has coquetted with or accepted these teachings, or of those pseudo-scientific works which have fostered them, will be found, we believe, in a clear knowledge of the goal to which they lead. To this end mainly we have given an outline of Madame Maria de Fos's work. If any further apology be required for polluting our pages with extracts from books of this class, we would say—adopting in great measure the language of an author, who, at the termination of last century, in consequence of an eruption of works of a like character, by English writers, immediately following the publication of Werther, was induced to discuss the subject of suicide:—"We consider ourselves free, and hold it a duty, to attack and censure in the most pointed terms such injudicious and mischievous publications, in which it has been endeavoured by false and specious glosses to lessen our horror at the crime, where no such indulgence was merited; and in which publications these dangerous encomiasts would fain make their heroes' or heroines' foibles, follies, and vices slide into virtues, or something very like them: and though a criminal indulgence of these passions, or the adoption of a vicious course, in order to avoid a real evil, was the manifest cause of their suicide, yet would endeavour to obscure our just abhorrence of vice and love of virtue under the deceitful covering of indiscriminate sensibility and compassion. Such glossers of vice deserve the severest reprehension, and cannot be too much exposed to shame and infamy."*

---

### Art. II.—THE BRITISH MEDICAL ASSOCIATION.

* *A Full Inquiry into the Subject of Suicide.* By Charles Moore, M.A., Rector of Caxton, &c. London, 1790.

